# Predicting Radiation Resistance in Breast Cancer with Expression Status of Phosphorylated S6K1

**DOI:** 10.1038/s41598-020-57496-8

**Published:** 2020-01-20

**Authors:** Jihye Choi, Yi Na Yoon, Nawon Kim, Chan Sub Park, Hyesil Seol, In-Chul Park, Hyun-Ah Kim, Woo Chul Noh, Jae-Sung Kim, Min-Ki Seong

**Affiliations:** 1 Department of Surgery, Korea Cancer Center Hospital, Korea Institute of Radiological and Medical Sciences, Seoul, Korea; 2 Department of Surgery, National Medical Centre, Seoul, Korea; 3 Division of Radiation Biomedical Research, Korea Institute of Radiological and Medical Sciences, Seoul, Korea; 4 Radiological and Medico-Oncological Sciences, University of Science and Technology, Daejeon, Korea; 5 Department of Pathology, Korea Cancer Centre Hospital, Korea Institute of Radiological and Medical Sciences, Seoul, Korea; 6 Division of Basic Radiation Bioscience, Korea Institute of Radiological and Medical Sciences, Seoul, Korea

**Keywords:** Predictive markers, Breast cancer

## Abstract

Emerging evidence suggests that the mammalian target of rapamcyin (mTOR) pathway is associated with radio-resistance in cancer treatment. We hypothesised that phosphorylated ribosomal S6 kinase 1 (p-S6K1), a major downstream regulator of the mTOR pathway, may play a role in predicting radio-resistance. Therefore, we evaluated the association of p-S6K1 expression with radio-resistance in breast cancer cell lines and patients. During median follow-up of 33 (range, 0.1–111) months for 1770 primary breast cancer patients who underwent surgery, patients expressing p-S6K1 showed worse 10-year loco-regional recurrence-free survival (LRFS) compared to that of p-S6K1-negative patients after radiotherapy (93.4% vs. 97.7%, *p* = 0.015). Multivariate analysis revealed p-S6K1 expression as a predictor of radio-resistance (hazard ratio 7.9, 95% confidence interval 1.1–58.5, *p* = 0.04). *In vitro*, CD44^high^/CD24^low^ MCF7 cells with a radioresistant phenotype expressed higher levels of p-S6K1 than control MCF7 cells. Furthermore, the combination of radiation with treatment of everolimus, an mTOR-S6K1 pathway inhibitor, sensitised CD44^high^/CD24^low^ MCF7 cells to a greater extent than MCF7 cells. This study provides *in vivo* and *in vitro* evidence for p-S6K1 expression status as an important marker for predicting the resistance to radiotherapy and as a possible target for radio-sensitization in breast cancer patients.

## Introduction

During the last few decades, advances in the molecular profiling of breast cancer have substantially changed treatment approaches towards personalised therapy, and provided patients with significantly improved survival rates and quality of life^1^. Despite these enormous strides in tailoring a variety of approaches to systemic therapies, the role of radiotherapy in precision medicine for breast cancer is only beginning to emerge^2–4^. Technical advances in radiotherapy have resulted in an improved ability to decrease dose delivery to surrounding vital organs such as the heart and lungs while accurately targeting the diseased tissue. In addition, a number of dose fractionation strategies have now been validated for patients with early- and advanced-stage breast cancer. Partial breast irradiation via external beam, brachytherapy, or intraoperative radiation techniques has been shown to limit the volume of irradiated tissue in selected patients while preserving efficacy^5–7^. However, in terms of biology, uniform doses of radiation have been typically delivered without consideration to differences across breast cancer subtypes. Consequently, the majority of breast cancer patients receive radiotherapy according to the type of surgery and clinical stage rather than based on their sensitivity to this kind of therapy.

Radiotherapy is a critical treatment modality for achieving loco-regional control in breast cancer patients. Moreover, the pivotal role of adjuvant radiotherapy in leading to reduced recurrence and long-term mortality has been well-established through the analyses of the Early Breast Cancer Trialists’ Collaborative Group^8,9^. Nevertheless, some patients still may not benefit from this treatment owing to individual variation in radio-sensitivity and may experience recurrences that ultimately challenge their prognosis and quality of life. It is therefore necessary to develop new biomarkers that predict the effectiveness of radiotherapy. Prediction of radio-resistance in individual patients may enable optimised dose modifications or application of radio-sensitisers to tumours with radio-resistance^2^. Although several tumour-related descriptive factors such as vascular invasion, larger tumour size, and higher grade have been considered as predictors of loco-regional recurrence to date, they provide limited information about the intrinsic tumour response to radiotherapy^10^.

Phosphorylated ribosomal S6 kinase 1 (p-S6K1) is a key downstream effector of the mammalian target of rapamycin (mTOR) pathway and has been considered an indirect marker of mTOR activity^11–13^. Because p-S6K1 relays many signals to promote further oncogenic translations in tumourigenesis^14^, upregulation of p-S6K1 in the tumour has been suggested to be predictive of resistance to systemic therapies such as endocrine therapy and chemotherapy in breast cancer patients^15–17^. However, few studies on p-S6K1 have focused on its role in the response to radiotherapy^18–20^. As mounting evidence indicates that the mTOR pathway is associated with the development radio-resistance in tumours^21–23^, as an indicator of mTOR activity p-S6K1 might be related with the development of radio-resistance in breast cancer.

Therefore, in this study, we investigated the association between the expression of p-S6K1 and radio-resistance in breast cancer patients and evaluated the clinical potential of p-S6K1 in predicting radio-resistance. Furthermore, we explored the association between p-S6K1 and radio-resistance in breast cancer stem cells.

## Results

### Patient characteristics

The median age of the patients included in this study was 51 years. The majority of patients who received radiotherapy (n = 1317, 74.4%) had breast conserving surgery (n = 1044, 79.3%), whereas the remainder underwent total mastectomy (Table 1). Positive and negative p-S6K1 expression was detected in 1323 (74.4%) and 447 (25.3%) patients, respectively. The p-S6K1 expression status was evenly distributed among patients treated with or without radiotherapy (*p* = 0.091). The numbers of loco-regional recurrences were also well distributed between the two groups (*p* = 0.094).

**Table 1:** Clinico-pathological characteristics of the 1770 patients.

		RT^b^ (+) (n = 1317)	RT (−) (n = 453)	p*
Age at diagnosis^a^	<50	701 (53.2%)	177 (39.1%)	<0.001
	≥50	616 (46.8%)	276 (60.9%)	
ER	Negative	420 (32.3%)	170 (37.9%)	0.032
	Positive	882 (67.7%)	278 (62.1%)	
PR	Negative	575 (44.2%)	245 (54.7%)	<0.001
	Positive	726 (55.8%)	203 (45.3%)	
HER2	Negative	966 (76.3%)	279 (64.6%)	<0.001
	Positive	300 (23.7%)	153 (35.4%)	
p-S6K1	Negative	319 (24.2%)	128 (28.3%)	0.091
	Positive	998 (75.8%)	325 (71.7%)	
Mass size	<2 cm	779 (59.1%)	258 (57.0%)	0.439
	≥2 cm	538 (40.9%)	195 (43.0%)	
Node status	Negative	792 (60.5%)	243 (54.0%)	0.017
	Positive	518 (39.5%)	207 (46.0%)	
Histology	IDC	1134 (86.1%)	395 (87.2%)	0.830
	ILC	59 (4.5%)	19 (4.2%)	
	Others	124 (9.4%)	39 (8.6%)	
Histologic grade	G1,2	777 (65.8%)	264 (65.2%)	0.427
	G3	403 (34.2%)	141(34.8%)	
Surgery	Breast conserving	1044(79.3%)	26(5.7%)	<0.001
	Total mastectomy	273(20.7%)	427(94.3%)	
Loco-regional recurrence	Negative	1279 (97.1%)	432 (95.4%)	0.094
	Positive	38 (2.9%)	21 (4.6%)	
Biologic subtype	Luminal A	746 (59.5%)	206 (48.6%)	<0.001
	Luminal B	168 (13.4%)	73 (17.1%)	
	HER2	130 (10.4%)	79 (18.5%)	
	Triple negative	209 (16.7%)	69 (16.2%)	

^a^Frequency (%).

^b^Radiotherapy.

*Pearson’s chi-square test and Fisher’s exact test.

During the median follow-up period of 33 months (range, 0.1–111 months), 167 recurrences and 53 deaths occurred. A total of 59 recurrences were classified as loco-regional recurrence involving 12 chest wall, 12 ipsilateral-breast, 15 axillary, 15 supraclavicular, and six internal mammary lymph node recurrences.

### Association of p-S6K1 expression status and loco-regional recurrence

**Figure 1:**
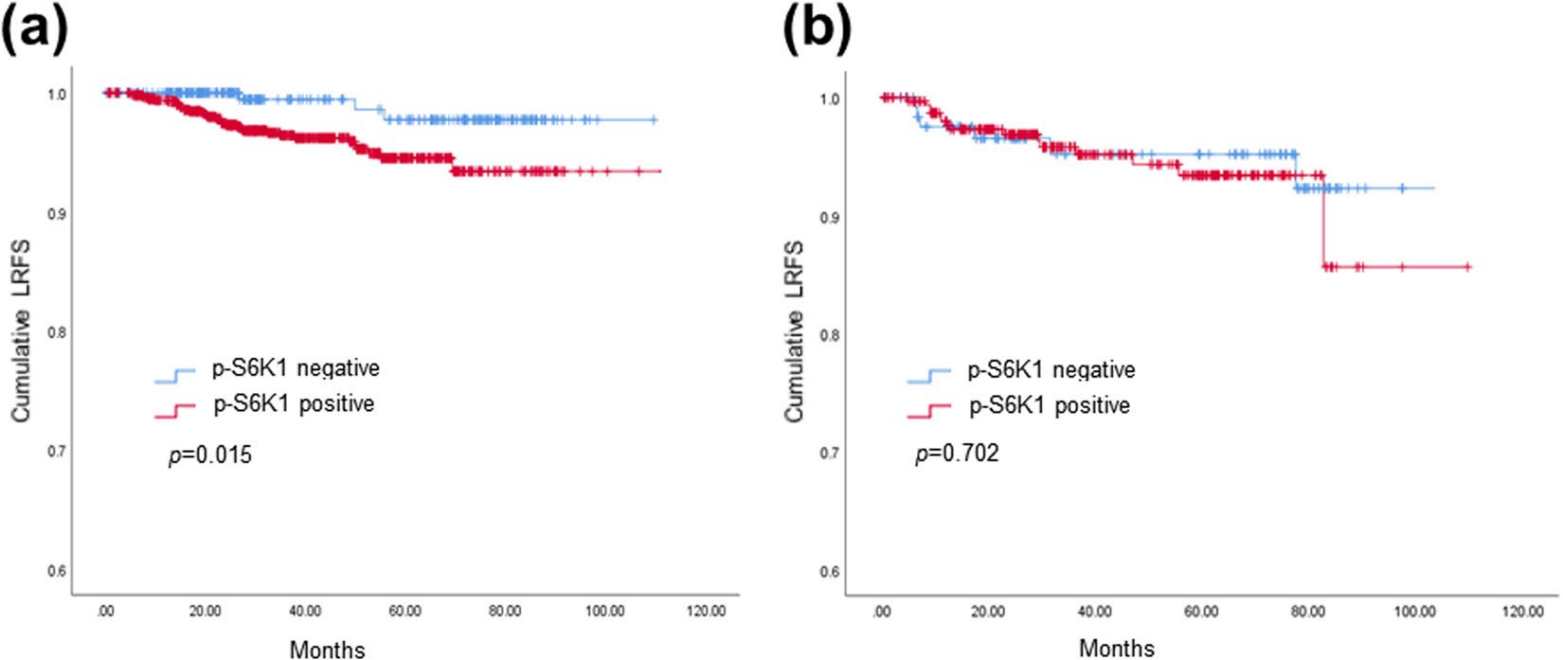
Loco-regional recurrence-free survival (LRFS) according to p-S6K1 expression status in patients with or without radiotherapy. Kaplan-Meier estimates of LRFS among patients treated with (**a**) and without (**b**) radiotherapy. Patients with positive p-S6K1 expression showed significantly worse LRFS compared to those with negative p-S6K1 expression when treated with radiotherapy (*p = 0.015). In contrast, no difference in LRFS based on p-S6K1 expression status was seen in patients who did not receive radiotherapy (*p = 0.702). *Kaplan-Meier survival estimate compared by a log-rank test.

Patients with positive p-S6K1 expression showed worse loco-regional recurrence-free survival (LRFS) compared to those with negative p-S6K1 expression when treated with radiotherapy. The 10-year LRFS rate in patients treated with radiotherapy was 93.4% for pS6K1-positive patients and was 97.7% for pS6K1-negative patients (*p* = 0.015, Fig. 1a). However, no association between p-S6K1 expression status and LRFS was observed when patients did not receive radiotherapy (*p* = 0.702, Fig. 1b). Loco-regional control rates at 10 years were 94.6% and 89.0% in patients with and without radiotherapy, respectively.

In univariate analysis to identify predictors of radio-resistance, positive p-S6K1 expression was found to be predictive of worse LRFS (*p* = 0.024) in patients treated with radiotherapy. After multivariate analysis, positive p-S6K1 expression (*p* = 0.044) remained a predictor of worse LRFS (Table 2). However, as mass size (*p* = 0.038) was also predictive of worse LRFS in the multivariate analysis, we cross-analysed mass size and p-S6K1 expression status. Ten loco-regional recurrences occurred in 779 patients with tumours ≤2 cm, and 28 recurrences occurred in 538 patients with tumours >2 cm (Supplementary Table S1). When the tumours were >2 cm, significantly worse LRFS was observed in patients with a positive p-S6K1 status compared to that of patients with a negative p-S6K1 status (*p* = 0.044). In contrast, there was no significant difference in LRFS according to p-S6K1 status in patients with tumours ≤2 cm (*p* = 0.202, Supplementary Fig. S1). However, among the 10 recurrences that occurred in tumours ≤2 cm, nine (90%) tumours were p-S6K1-positive, whereas only one (10%) tumour had a negative p-S6K1 status.

**Table 2:** Univariate and multivariate analyses on potential predictors of radio-resistance (n = 1317).

Variables	Univariate analysis	p*	Multivariate analysis	p*
	Hazard Ratio (95% CI)		Hazard Ratio (95% CI)	
Age				
≤50 vs >50	1.48(0.78–2.80)	0.234	1.82 (0.86–3.87)	0.120
ER status				
(−) vs (+)	0.31(0.16–0.60)	<0.001	0.48 (0.20–1.16)	0.105
PR status				
(−) vs (+)	0.57(0.30–1.09)	0.088	0.90 (0.38–2.14)	0.818
HER2 status				
(−) vs (+)	3.47(1.77–6.80)	<0.001	2.00 (0.94–4.28)	0.074
Size of tumour				
≤2 cm vs >2 cm	4.24(2.06–8.73)	<0.001	2.77 (1.06–7.23)	0.038
Node status				
(−) vs (+)	2.65(1.37–5.13)	0.04	2.34 (0.95–5.77)	0.660
Histologic grade				
1 or 2 vs 3	2.37(1.17–4.80)	0.017	1.0 (0.45–2.22)	1.000
p-S6K1				
(−) vs (+)	3.91(1.20–12.78)	0.024	7.86 (1.06–58.47)	0.044

*Cox proportional hazards model.

### Increased p-S6K1 could be associated with radio-resistance in breast cancer stem cells

**Figure 2:**
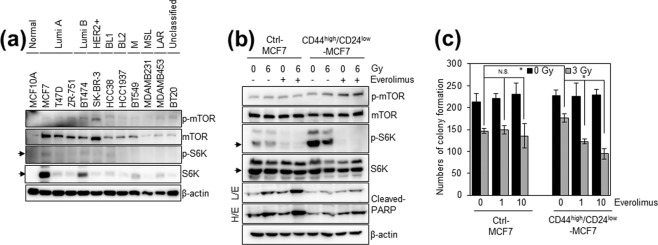
Increased levels of p-S6K1 are associated with radio-resistant CD44^high^/CD24^low^ MCF7 cells. (**a**) Cells were analysed with immunoblotting and the indicated antibodies. (**b**) and (**c**) Control MCF7 cells and CD44^high^/CD24^low^ MCF7 cells were pre-treated without or 10 nM everolimus and then treated with 0, 3, or 6 Gy irradiation for 42 h. Cells were analysed with immunoblotting and the indicated antibodies (**b**). The number of colonies was measured (**c**). Data represent typical results and are presented as the mean ± standard deviation of three independent experiments. Arrows indicate the signal from S6K1 and p-S6K1. Blotting results in (**a**) and (**b**) were cropped from different gels and are therefore delineated by white spaces and lines. The original blot data are shown in Supplementary Figures S3. N.S.: not significant; H/E: high exposure; L/E: low exposure. *p < 0.01 based on a paired t-test.

Because our clinical analysis showed a positive correlation between p-S6K1 expression and radio-resistance in breast cancer patients, we further examined whether the S6K1 pathway regulates the radio-resistance of breast cancer *in vitro*. First, we determined the expression levels of phosphorylated-mTOR (p-mTOR), a direct upstream regulator of S6K1, and p-S6K1 in normal breast cells and several breast cancer cell lines. We found that the phosphorylated and endogenous levels of mTOR and S6K1 were generally increased in breast cancer cells compared to those of normal breast MCF10A cells (Fig. 2a). Of the breast cancer cell lines evaluated, MCF7 cells expressed higher levels of phosphorylated and endogenous mTOR and S6K1 (Fig. 2a). Furthermore, we found that CD44^high^/CD24^low^ MCF7 cells, a breast cancer stem cell line with a radioresistant phenotype^24,25^ (Supplementary Fig. S2), expressed higher levels of p-S6K1 compared to those of control MCF7 cells (Fig. 2b), suggesting that high expression levels of p-S6K1 are involved in the radio-resistance of breast cancer. Thus, control and CD44^high^/CD24^low^ MCF7 cells were used to further examine whether the mTOR-S6K1 pathway regulates the radio-resistance of breast cancer. Notably, we found that combining radiation with treatment of everolimus, a clinically available inhibitor of the mTOR-S6K1 pathway^26^, sensitised the radio-resistant CD44^high^/CD24^low^ MCF7 cells to a greater degree than observed in the control MCF7 cells (Fig. 2b,c). Therefore, our data suggest that increased levels of p-S6K1 expression could be associated with radio-resistance in breast cancer stem cells.

## Discussion

In this study, we provide *in vitro* and clinical evidence that p-S6K1 expression status could be associated with a reduced response to radiotherapy in breast cancer. To the best of our knowledge, our study is the first to demonstrate the potential of p-S6K1 expression status as a marker for radio-resistance in breast cancer. To date, only a few studies have provided indirect evidence that S6 kinase may be associated with the response to radiotherapy. However, our analysis directly compared the outcomes of radiotherapy according to S6K1 expression status.

In a multi-centre study conducted by van der Hage *et al*. in 2004^19^, the predictive potential of S6K1 upregulation was evaluated in premenopausal, node-negative early breast cancer patients. The authors showed that S6K1 upregulation was associated with poor loco-regional control in overall patients [hazard ratio (HR): 2.67, 95% confidence interval (CI): 1.39–5.14, *p* = 0.003] and in patients who received breast conserving surgery (HR 2.83, 95% CI: 1.42–5.62, *p* = 0.003), which comprised 80% of the total study population. Although it could be inferred that S6K1 upregulation is related to the response to radiotherapy because patients are likely to be treated with radiotherapy after breast conserving surgery, the role of radiotherapy was not examined in their study^19^. In another study conducted by Perez-Tenorio *et al*. in 2011^20^, S6K1 and S6K2 gene amplification was assessed in postmenopausal breast cancer patients. One of the aims of the study was to examine the clinical potential of both genes as predictive factors regarding radiotherapy, and the sub-analysis indicated that, in contrast to tumours with S6K1 amplification, tumours with a normal S6K1 gene copy number responded better to radiotherapy compared to chemotherapy (HR 0.27, 95% CI: 0.11–0.66, *p* = 0.0038)^20^. Although the authors suggested that S6 kinases may play a role in predicting the response to radiotherapy, the study could not compare radiotherapy versus none. In addition, a preclinical study conducted in 2006 supported a unique role of p-S6K1 in developing radio-resistance in breast cancer^18^. The authors showed that ionizing radiation increases both mTOR signalling and subsequent p-S6K1 levels in breast cancer cells. However, independent regulation of p-S6 ribosomal protein levels was observed over time despite mTOR activation^18^, implying that there may be other radiation-induced pathways regulating the phosphorylation of S6K1^12,27,28^. Consequently, in addition to simply acting as a downstream effector of the mTOR pathway, p-S6K1 may provide additional predictive information on radiation-mediated changes.

Our results provide additional evidence to support the aforementioned studies that the expression status of p-S6K1 could be a potential marker for radio-resistance in breast cancer. Although positive p-S6K1 expression appeared to be a better predictor of radio-resistance in patients with tumours >2 cm than in those with tumours ≤2 cm, we suggest that p-S6K1 status might nevertheless be associated with radio-resistance in tumours ≤2 cm based on the results of our experimental study. In addition, since our data were not derived from a matched set with respect to tumour size, the low number of recurrence events (n = 10) in patients with tumours ≤2 cm may have resulted in the insignificant *p*-value in this group. It is possible that the naturally excellent prognosis of this patient group (T1 treated with radiation therapy) was insufficient to detect a significant LRFS difference (5-year LRFS 98.7% vs. 97.4% for p-S6K1-negative and -positive patients, respectively; data not shown). Nevertheless, among the 10 recurrences, the proportion of tumours with positive p-S6K1 expression was exclusively higher (n = 9, 90%) than that for patients with a negative p-S6K1 status (10%), thereby supporting our hypothesis.

Moreover, although the role of p-S6K1 needs to be further explored, we suggest that the study of p-S6K1 may provide insights into the development of novel radio-sensitising agents in breast cancer patients. Increased recognition of the importance of the mTOR pathway in cancer treatment has called for exploration of mTOR inhibitors as radio-sensitisers for treating many types of cancer^29–31^. Nevertheless, studies on breast cancer in view of p-S6K1 are scarce. We found only one study in which pre-treatment with everolimus, a clinical mTOR inhibitor, radio-sensitised MDA-MB-231 and MCF7 breast cancer cells by blocking radiation-induced mTOR-S6K1 signalling^18^. The salient point of our experiments is that we chose to compare the expression levels of p-S6K1 in breast cancer stem cells with a radio-resistant phenotype (CD44^high^/CD24^low^ MCF7 cells) to that of MCF7 breast cancer cells. Cancer stem cells are largely responsible for the development of radio-resistance during treatment because they have the potential to overcome the anti-cancer effects of radiotherapy and contribute to the generation of a radio-resistant heterogeneous tumour cell population^32–34^. The prominently increased p-S6K1 levels in radio-resistant breast cancer stem cells and distinct reversal of these levels by combining radiation and everolimus compared to control MCF cells indicate that p-S6K1 is at least partially responsible for treatment failure, and that inhibition of the mTOR-S6K1 pathway may provide an additional targeted approach to improve the outcome of radiotherapy.

A limitation of our work is that it was a single centre-based retrospective study. In addition, as there are no international standards guiding the staining and stratification of p-S6K1 expression levels, careful interpretation is required to avoid bias. The incidence of high p-S6K1 expression varies among studies, as different criteria have been used^16,19,35–37^. Although upregulation of S6K1 expression is found in 10–30% of primary breast cancers, positive p-S6K1 expression was observed in approximately 75% of the samples in our study. Therefore, further prospective research in other populations is warranted to validate the general applicability of these results.

The strengths of our study are that we stratified relatively large numbers (n = 1770) of patients based on the receipt of radiotherapy to explicitly analyse the clinical association between p-S6K1 levels and radio-resistance. We demonstrated that p-S6K1could predict radio-resistance in breast cancer. In addition, this study is one of the few experimental studies to demonstrate the possibility of using S6K1 as a target for radio-sensitisation in breast cancer.

To date, several markers such as the cytoplasmic expression of peroxiredoxin-I and circulating tumour cells have been identified to correlate with the radiotherapy response in breast cancer^3,38,39^. However, most of these markers require a translational process for acquisition or interpretation and none is currently used to guide treatment decisions in clinical practice. Nevertheless, as p-S6K1 levels can be easily assessed with immunohistochemical staining owing to commercially available antibodies, they could be readily utilised in a clinical setting for predicting radio-resistance. A better understanding of the roles of p-S6K1 expression through experimental studies and accurate prediction of radio-resistance may provide a basis for its application in individualised radiotherapy in the future.

## Conclusion

p-S6K1 expression status was associated with a reduced response to radiotherapy in patients with breast cancer. This indicates the potential of p-S6K1 levels as a predictor of the response to radiotherapy and as a novel target for radio-sensitisation to increase the efficacy of radiotherapy.

## Methods

### Study design and patient selection

A total of 1770 patients who received curative surgery for primary breast cancer between March 2008 and December 2015 were included in this study. Patients with *in situ* carcinoma and with a diagnosis of other primary malignancies were excluded. Radio-resistance was defined as having acquired loco-regional recurrence after completion of adjuvant radiotherapy. Information on patient age (<50 or ≥50 years), tumour size (<2 cm or ≥2 cm), nodal status (positive or negative), hormone receptor status, human epidermal growth factor receptor (HER)-2 status, histologic grade (1 and 2 or 3), type of surgery, and p-S6K1 status were reviewed retrospectively from our database, a web-based system that has been used to collect information on breast cancer patients since 1983. To date, more than 17,000 breast cancer patients have been registered in this database. The requirement for informed consent was waived owing to the retrospective nature of this study by the institutional review board of Korea Cancer Centre Hospital that approved the protocols of this study [2018-03-012]. All procedures performed in studies involving human participants were conducted in accordance with the ethical standards of the institutional review board of Korea Cancer Centre Hospital and with the 1964 Helsinki Declaration and its later amendments or comparable ethical standards. The design and data reporting of this study are in line with the REMARK criteria for data reporting^40^.

### Immunohistochemical staining

**Figure 3:**
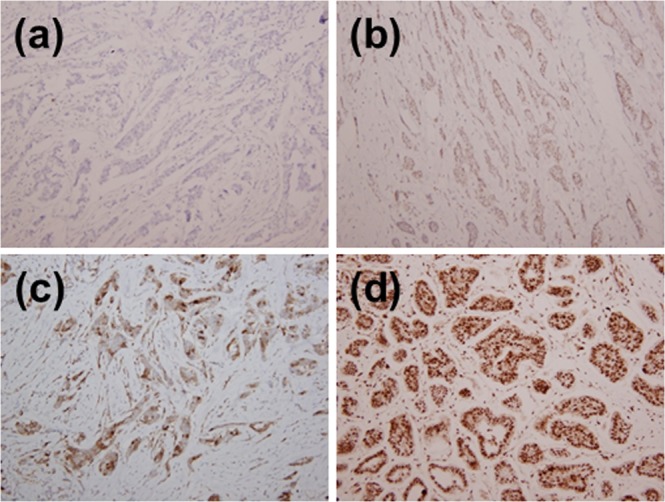
Immunohistological staining of p-S6K1 protein (100 × magnification). (**a**) Tumour with a negative score. (**b**) Tumour with a score of 1+. (**c**) Tumour with a score of 2+. (**d**) Tumour with a score of 3+.

Routine immunohistochemical assessment of oestrogen receptor (ER), progesterone receptor (PR), HER-2, and p-S6K1 expression was performed after the acquisition of each specimen at the diagnosis of breast cancer prior to surgery. Formalin fixed, paraffin-embedded tumour tissue blocks of core needle biopsied specimens were preferentially used^41^. However, tissue from post-surgical specimens was used when core needle biopsied specimens were unavailable. ER positivity was determined as the expression of ER detected in at least 1% of tumour cells as determined by immunohistochemistry. HER-2 upregulation was determined as a score of 3+ in immunohistochemical staining or a score of 2+ with positive gene amplification in *in situ* hybridisation. The p-S6K1 expression status was evaluated with immunohistochemistry and scored in a range from 0 to 3+, with a mouse monoclonal antibody against p-S6K1 (Cell Signaling Technology, Danvers, MA, USA; dilution 1:50). A score of 0 was defined as p-S6K1-negative, whereas scores from 1+ to 3+ were considered as p-S6K1-positive, with higher values indicating increased expression levels of p-S6K1. Details of the procedures are described in our previous report, in which we successfully evaluated the p-S6K1 status of 304 breast cancer patients^17^. Examples of positive and negative p-S6K1 expression on immunohistochemical staining are shown in Fig. 3.

### Statistical analysis

Statistical analyses were performed using SPSS version 25 (SPSS, Chicago, IL, USA). Chi-square and Fisher’s exact tests were employed to investigate the correlation between the clinico-pathological parameters and p-S6K1 expression status in each group. LRFS was defined as the time from the diagnosis of primary breast cancer to the time of first detection of loco-regional recurrence by physical examination or radiological imaging. Loco-regional recurrences presenting simultaneously with or after the diagnosis of distant metastasis were included. Kaplan-Meier plots with log-rank tests were used to analyse differences in survival between groups. Univariate and multivariate analyses were performed with the Cox-proportional hazard regression model to calculate HR and 95% CI values. A *p*-value of <0.05 was considered statistically significant.

### Cell culture and treatment

All cell lines were purchased from American Type Culture Collection (Manassas, VA, USA). MCF7, T47D, ZR-751, BT474, SKBR3, MDA-MB-453, and MDA-MB-231 cells were cultured in Dulbecco’s modified Eagle medium (DMEM; Corning, NY), and HCC1937, HCC38, and BT20 cells were cultured in RPMI medium (Corning). MCF10A cells were maintained in DMEM/F12 (Invitrogen, CA, USA) supplemented with 20 ng/mL epidermal growth factor (Peprotech, London, UK), 0.5 mg/mL hydrocortisone (Sigma-Aldrich, St. Louis, MO, USA), 100 ng/mL cholera toxin (Sigma-Aldrich), and 10 μg insulin (Sigma-Aldrich). All media were supplemented with 10% foetal bovine serum (Corning) and 1% penicillin/streptomycin, and maintained in a humidified 5% CO_2_ incubator at 37 °C. Radioresistant CD44^high^/CD24^low^ MCF7 cells were established using a previously described method^24,25^. In brief, CD44^+^/CD24^−^ subpopulations from MCF7 cells were isolated according to their surface markers with flow cytometry using a FACS Aria II system (BD Biosciences, San Diego, CA, USA). A radioresistant phenotype was determined using colony and sphere forming assays in response to irradiation. Cells were irradiated using a ^137^cesium (Cs) ray source (Atomic Energy of Canada, Mississauga, Canada) at a dose rate of 3.81 Gy/min. Everolimus (10 nM; Selleckchem, Houston, TX, USA) was used to inhibit the mTOR-S6K1 pathway.

### Clonogenic assay

Cell survival after irradiation was determined by a clonogenic assay as described previously^25,42^. In brief, 500 MCF7 or CD44^high^/CD24^low^ MCF7 cells were seeded in triplicate in 60-mm tissue culture dishes and treated with the indicated conditions without or with 3- or 6-Gy radiation using Biobeam GM8000 (Gamma-Service Medical GmbH, Leipzig, Germany). After 10–14 days, colonies were fixed with methanol, stained with 0.5% crystal violet, and counted using ImageJ software (https://imagej.nih.gov).

### Western blotting analysis

Western blotting was performed in accordance with a previously described protocol^42,43^. In brief, proteins were separated using sodium dodecyl sulphate-polyacrylamide gel electrophoresis, transferred to a polyvinylidene fluoride membrane, and detected through incubation with specific antibodies. The following antibodies were used: anti-phospho-S6K1 (9205; Cell Signaling Technology, MA, USA), anti-S6K1 (SC230; Santa Cruz Biotechnology, CA, USA), anti-phospho-mTOR (2971; Cell Signaling Technology), anti-mTOR (AP6273C; Abgent, CA, USA), anti-phospho(Thr705)-STAT3 (9145; Cell Signaling Technology), anti-HER2 (2165; Cell Signaling Technology), and anti-β-actin (AM1021B; Abgent). Blots were developed using a peroxide-conjugated secondary antibody and an enhanced chemiluminescence detection system (GE Healthcare Life Science, Little Chalfont, UK). Images were obtained using an Amersham Imager 600 system (GE Healthcare Life Science).

## Supplementary information

Supplementary Information

## Data Availability

Most of the data generated or analysed during this study are included in this published article and supplementary materials. The datasets generated during the study are available from the corresponding author on reasonable request.
