# Supplementary material for: Predicting Radiation Resistance in Breast Cancer with Expression Status of Phosphorylated S6K1
Source: Sci Rep. 2020 Jan 20;10:641. doi: 10.1038/s41598-020-57496-8 (PMC6971275; doi:10.1038/s41598-020-57496-8)
Supplement: Supplementary file 1 — Supplementary Information [file 41598_2020_57496_MOESM1_ESM.pdf]

Supplementary Materials

Title: Predicting Radiation Resistance in Breast Cancer with Expression Status of Phosphorylated S6K1

Authors and affiliations: Jihye Choi[1,2]†, Yi Na Yoon[3, 4]†, Nawon Kim[1], Chan Sub Park[1], Hyesil Seol[5], In-Chul Park[6], Hyun-Ah Kim[1], Woo Chul Noh[1], Jae-Sung Kim[3,4*], Min-Ki Seong[1*]

[1]Department of Surgery, Center Hospital, Korea Institute of Radiological and Medical Sciences, Seoul, Korea

[2]Department of Surgery, National Medical Center, Seoul, Republic of Korea
[3]Division of Radiation Cancer Research, Korea Institute of Radiological and Medical Scien ces, Seoul, Korea
[4]Radiological and Medico-Oncological Sciences, University of Science and Technology, Daejeon, Korea
[5]Department of Pathology, Korea Cancer Center Hospital, Korea Institute of Radiological and Medical Sciences, Seoul, Korea

[6]Division of Basic Radiation Bioscience, Korea Institute of Radiological and Medical Sciences, Seoul, Republic of Korea

†These authors have contributed equally to this work

Keywords: Radiation resistance; Breast cancer; S6K1 (ribosomal S6 Kinase 1); Immunohistochemistry; Prognostic markers; Recurrence

Financial support: This study was supported by a grant of the Korea Institute of Radiological and Medical Science (KIRAMS), funded by Ministry of Science, ICT and Future Planning, Republic of Korea. (50472-2019, 50531-2019)

Conflict of interest: The authors declare no potential conflicts of interest.

Corresponding authors: Min-Ki Seong MD. Department of Surgery, Korea Cancer Center Hospital, Korea Institute of Radiological & Medical Sciences, 75 Nowon-ro Nowon-ku, Seoul 130-706, Korea. Tel.: +82-2-970-1251; Fax: +82-2-970-2419; E-mail: mailto:mklegend@kcch.re.kr or Jae-Sung Kim Ph.D. Division of Radiation Cancer Research, Korea Institute of Radiological and Medical Sciences, 215-4 Gongneung-Dong, Nowon-Ku, Seoul 139-706, Korea. Tel: +82-2-970-1669, Fax: +82-2-970-2417, E-mail: jaesung@kirams.re.kr

Supplementary Figures

Supplementary figure S1. Loco-regional recurrence free survival (LRFS) according to p-S6K1 expression status in patients with tumors > 2cm and ≤ 2cm. (a) Patients with tumors >2cm. (b) Patients with tumors ≤ 2cm. Kaplan-Meier estimates of LRFS among patients treated with tumors > 2cm (a) and ≤ 2cm (b). When tumor size was >2cm, worse LRFS was seen in patients with positive p-S6K1 status compared to those with negative p-S6K1 status, and the difference was statistically significant (*p* =0.044). In contrast, in patients with tumors ≤ 2cm, there was no difference in LRFS according to p-S6K1 status (*p** = 0.202) *Kaplan-Meier survival estimate compared by a long-rank test.

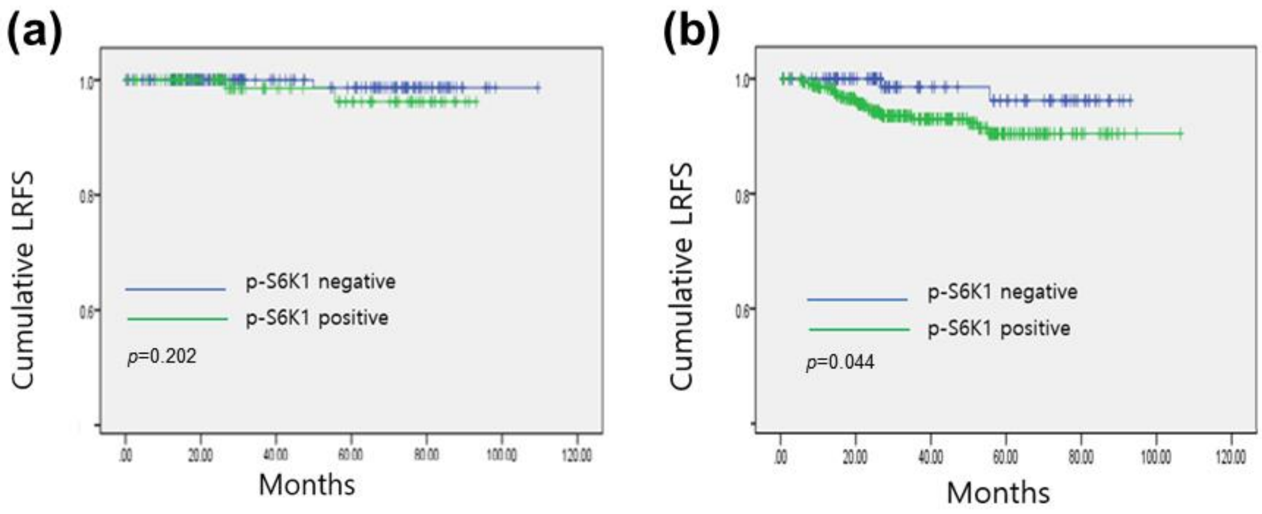

Supplementary figure S2. Charaterization of $CD44^{high}$/$CD24^{low}$ MCF7 cells with radioresistant phenotype. (a) The flow cytometric analysis of MCF7 cells labeled without or with CD44-FITC and CD24-PE antibodies. The total MCF7 cells were divided into two subpopulations: CD44high/CD24low and CD44low/CD24low. (b) The sphere forming assay was performed with MCF7 and sorted MCF7 (CD44high/CD24low) cells. Representative images of the sphere forming assay in MCF7 and sorted MCF7 cells (left panel). Scale bars, 100 µm. The sphere forming capacity was measured by the sphere diameter (µm) (right panel). (c) MCF7 and sorted MCF7 cells were analyzed by immunoblotting with the indicated antibodies. (d) MCF7 and sorted MCF7 cells were irradiated with 3 Gy and then we performed a colony formation assay. The data represent typical results and were presented as the mean ± standard deviation of three independent experiments; $p < 0.01$ (**) and $p < 0.05$ (*). *Paired t-test.

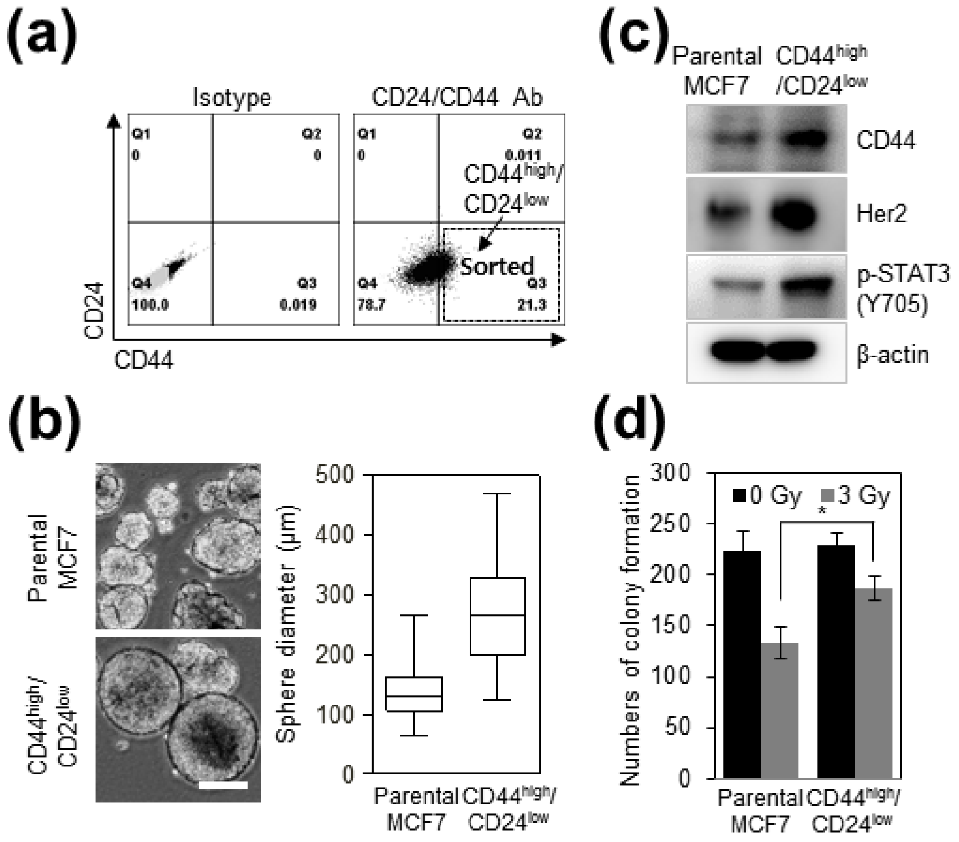

Supplementary figure S3. Original blots used for Figure 2 (a).

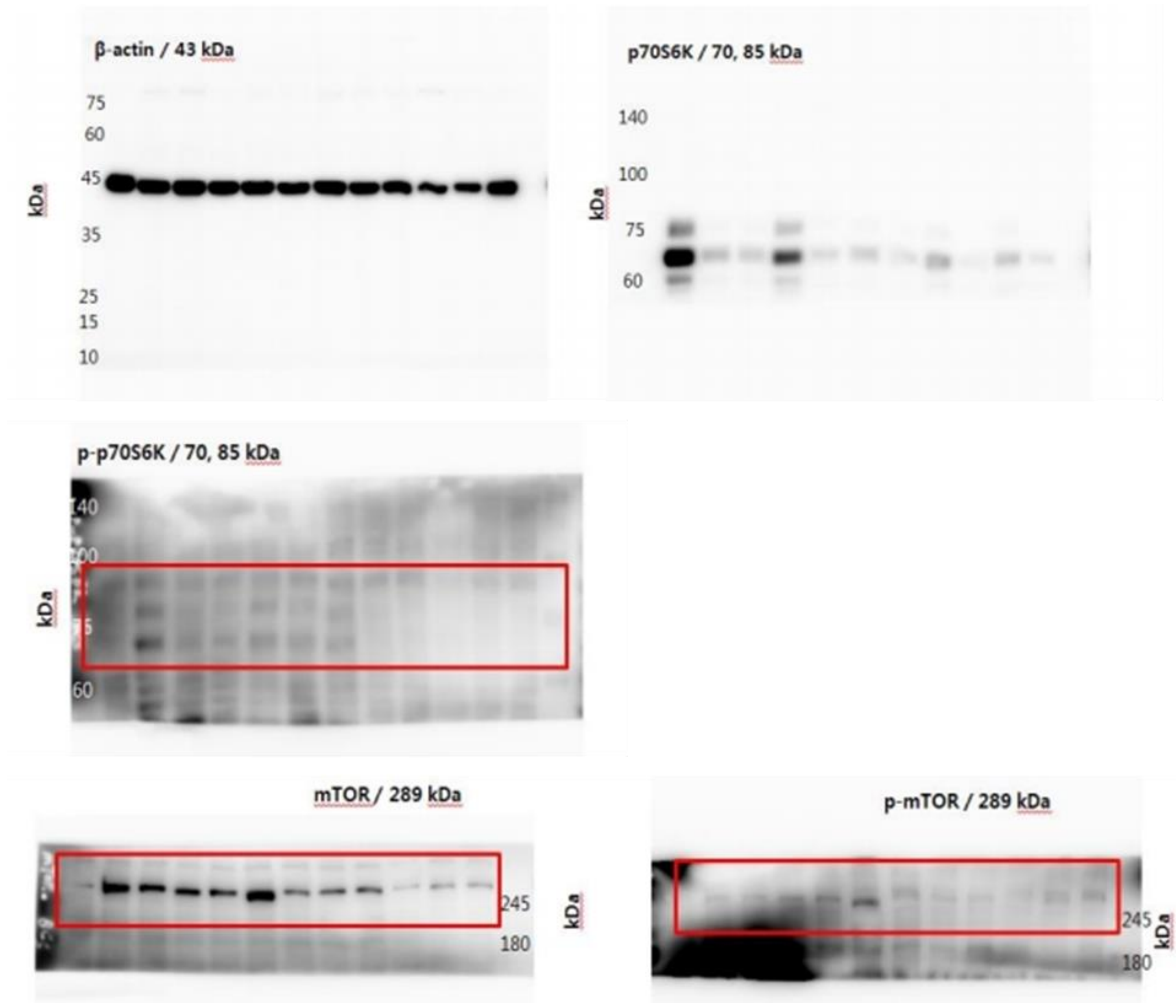

Original blots used for Figure 2 (b).

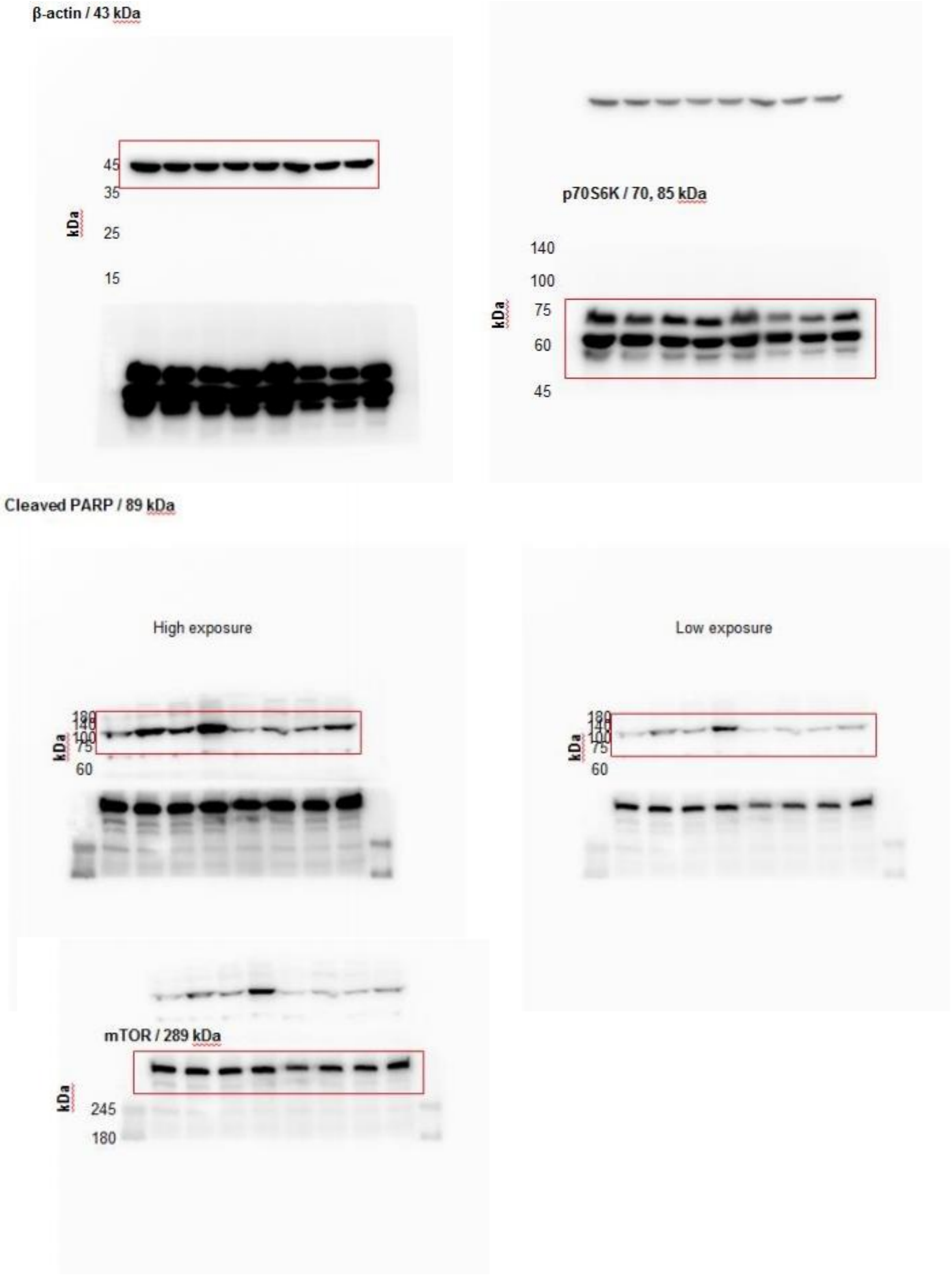

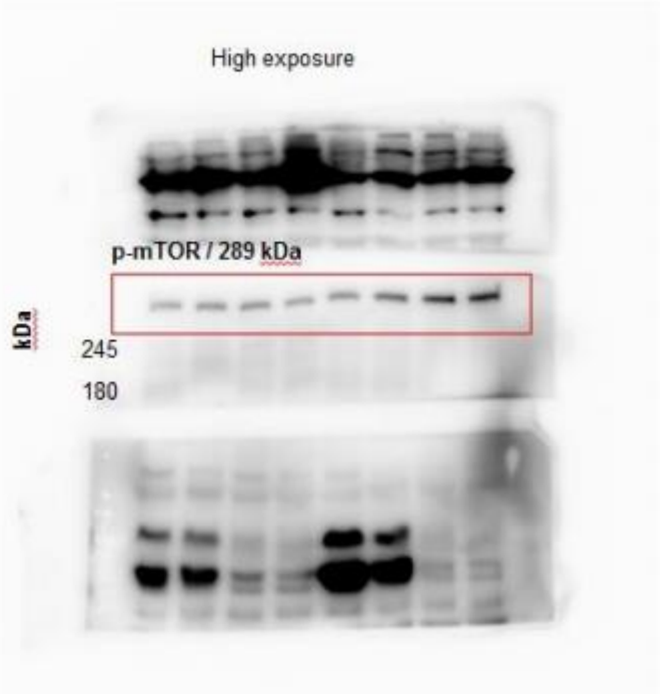
High exposure
p-mTOR / 289 kDa
kDa
245
180

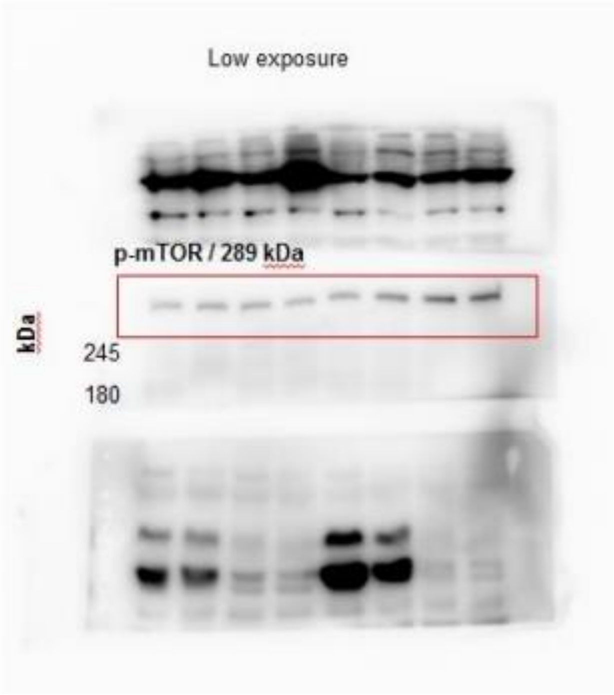
Low exposure
p-mTOR / 289 kDa
kDa
245
180

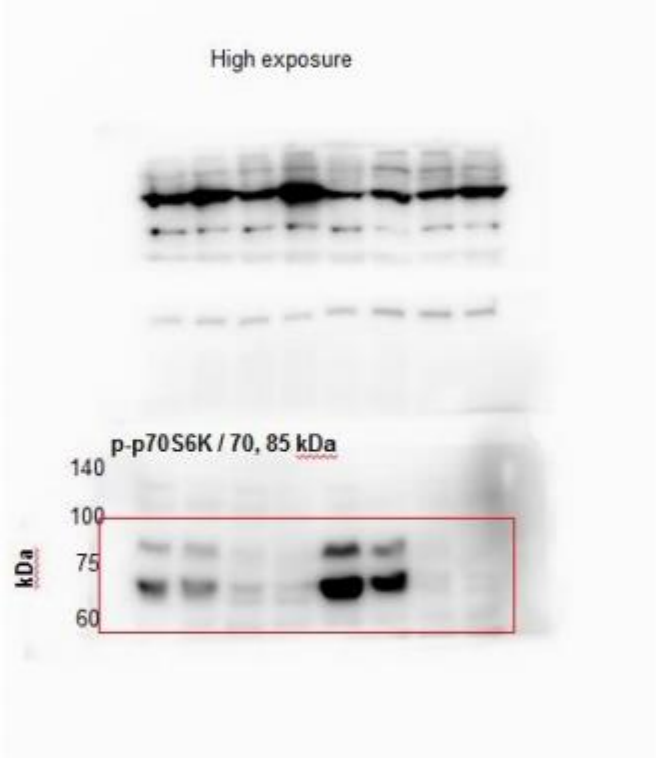
High exposure
p-p70S6K / 70, 85 kDa
140
100
75
60
kDa

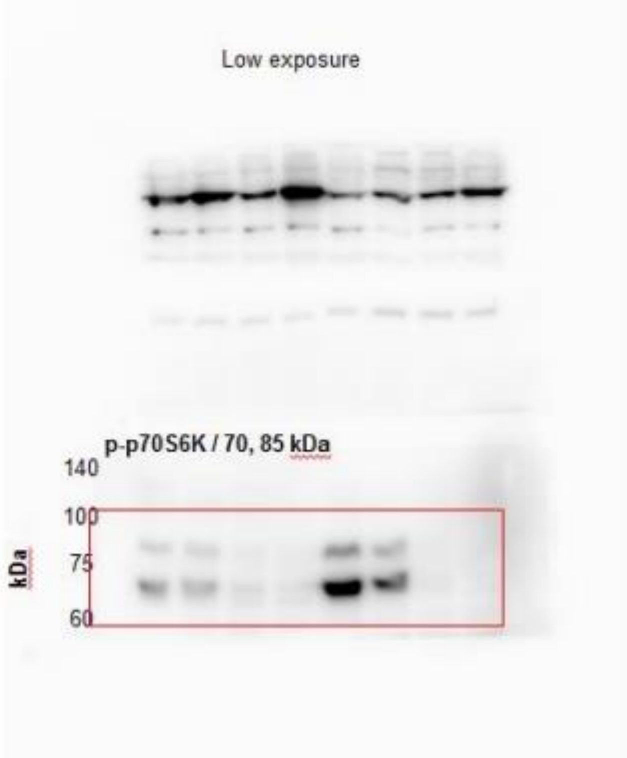
Low exposure
p-p70S6K / 70, 85 kDa
140
100
75
60
kDa

Original blots used for supplementary Figure S2 (c).

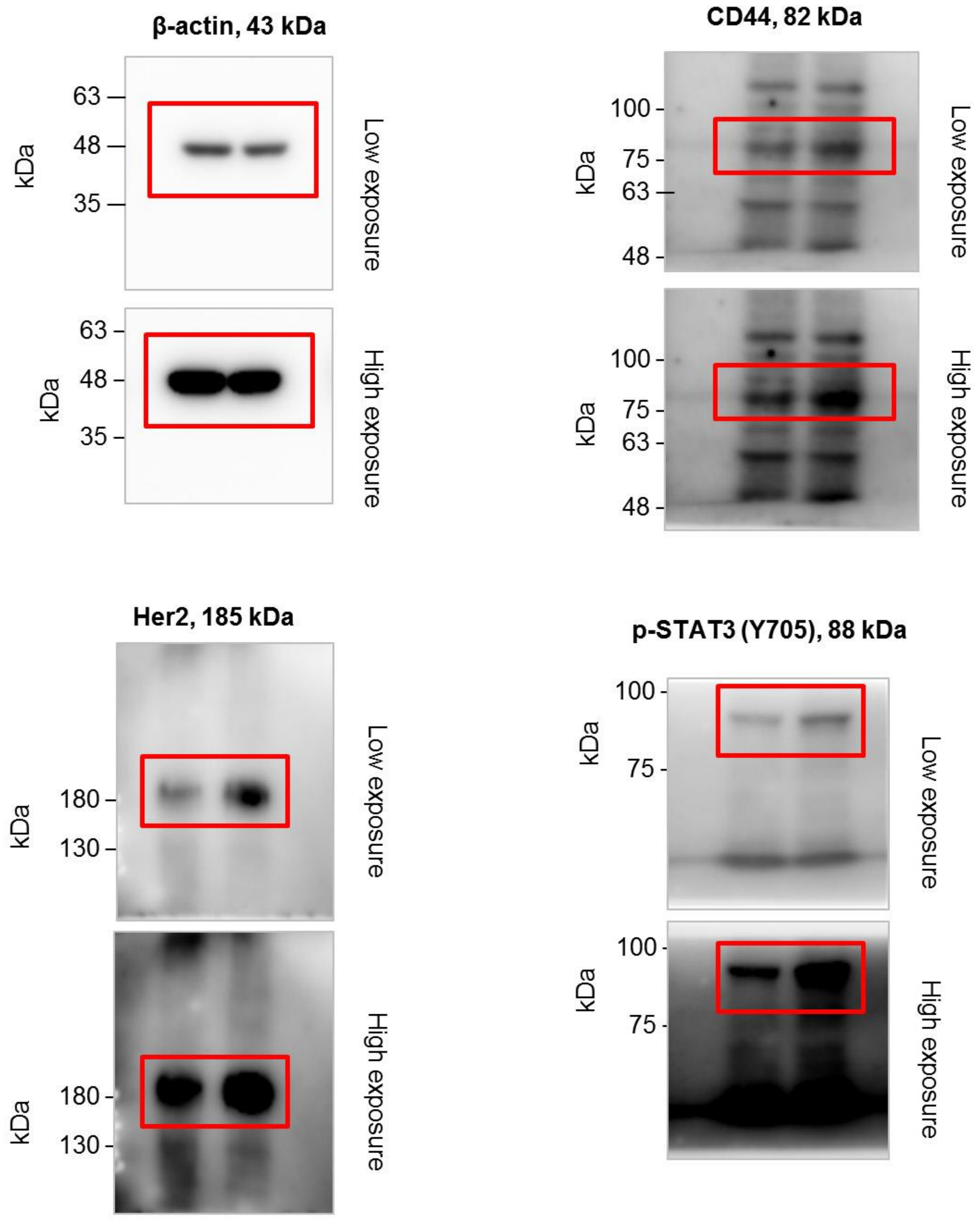

Supplementary Table1. Patient characteristics according to mass size and p-S6K1 status (n=1317)

		Mass size ≤2cm (n=779)			Mass size >2cm (n=538)			
		p-S6K1 positive	p-S6K1 negative	*P**	p-S6K1 positive	p-S6K1 negative	*P**	Overall *p**
Age at diagnosis	<50	277 (71.8%) [a]	109 (28.2%)	0.140	239 (75.9%)	76 (24.1%)	0.169	0.053
	≥50	301 (76.6%)	92 (23.4%)		181 (81.2%)	42 (18.8%)		
Node stage	Negative	155 (25.2%)	459 (74.8%)	0.541	47 (26.4%)	131 (73.6%)	0.096	0.187
	Positive	44 (27.8%)	114 (72.2%)		71 (19.7%)	289 (80.3%)		
ER	Negative	148 (74.0%)	52 (26.0%)	1.000	171 (77.7%)	49 (22.3%)	1.000	0.890
	Positive	422 (74.0%)	148 (26.0%)		243 (77.9%)	69 (22.1%)		
PR	Negative	206 (71.3%)	83 (28.7%)	0.203	218 (76.2%)	68 (23.8%)	0.402	0.194
	Positive	364 (75.7%)	117 (24.3%)		195 (79.6%)	50 (20.4%)		
HER2	Negative	441 (73.6%)	158 (26.4%)	0.091	279 (76.0%)	88 (24.0%)	0.079	0.008
	Positive	120 (80.5%)	29 (19.5%)		126 (83.4%)	25 (16.6%)		
Histologic grade	G1orG2	390 (72.9%)	145 (27.1%)	0.313	180 (74.4%)	62 (25.6%)	1.000	0.023
	G3	129 (77.2%)	38 (22.8%)		191 (80.9%)	45 (19.1%)		
Locoregional recurrence	Negative	569 (74.0%)	200 (26.0%)	0.467	394 (77.3%)	116 (22.7%)	0.060	0.019
	Positive	9 (90.0%)	1 (10.0%)		26 (92.9%)	2 (7.1%)		

ER: estrogen receptor, PR: progesterone receptor, HER2: human epidermal growth factor receptor
a Frequency (%)
*Pearson’s chi-square test
